# Accommodative and Vergence Responses to a Moving Stimulus in Concussion

**DOI:** 10.1167/iovs.65.12.45

**Published:** 2024-10-30

**Authors:** Jennifer X. Haensel, Sophia Marusic, Kristin E. Slinger, Carissa H. Wu, Neerali Vyas, Christabel A. Ameyaw Baah, Amber Hu, Joellen Leonen, Caitlyn Y. Lew, Gayathri Srinivasan, Amir Norouzpour, Erin Jenewein, Siva Meiyeppen, Mitchell Scheiman, Aparna Raghuram, Tawna L. Roberts

**Affiliations:** 1 Spencer Center for Vision Research, Byers Eye Institute at Stanford University, Palo Alto, California, United States; 2 Boston Children's Hospital, Harvard Medical School, Boston, Massachusetts, United States; 3 Pennsylvania College of Optometry, Salus University, Elkins Park, Pennsylvania, United States

**Keywords:** accommodation, vergence, concussion, adolescence, photorefraction

## Abstract

**Purpose:**

Concussed adolescents often report visual symptoms, especially for moving targets, but the mechanisms resulting in oculomotor deficits remain unclear. We objectively measured accommodative and vergence responses to a moving target in concussed adolescents and controls.

**Methods:**

Thirty-two symptomatic concussed participants (mean age, 14.4 ± 2.6 years; mean days since concussion, 107 days; range, 36–273 days) and 32 healthy controls (mean age, 12.7 ± 2.1 years) viewed a movie binocularly (closed-loop) and monocularly (vergence open-loop), as well as a Difference of Gaussians (DoG) target binocularly (accommodation open-loop). The movie or DoG target sinusoidally moved toward and away from participants at a 0.1-hertz (Hz) frequency at four separate stimulus amplitudes (1.50 diopters [D], 1.00 D, 0.50 D, 0.25 D) around a 2.50-D midpoint. Accommodation and vergence were continuously measured at 50 Hz using the PowerRef 3. Fourier analysis was used to assess the response amplitudes at the 0.1-Hz frequency. A 2 × 3 analysis of variance with the factors group (concussed, control) and viewing condition (binocular, monocular, DoG) was conducted on response amplitudes.

**Results:**

Across groups, accommodative and vergence responses were significantly higher in binocular than monocular conditions (*P* < 0.001), but not DoG conditions. Compared to controls, concussed participants had significantly reduced monocular accommodative responses (*P* < 0.012; e.g., at 1.50 D, controls = 1.09 ± 0.47 D and concussed = 0.80 ± 0.36 D, *P* = 0.011). No group differences were observed for vergence responses in any viewing condition.

**Conclusions:**

Accommodative and vergence responses to the moving target were largely driven by disparity cues for both groups, with only minimal improvements in the presence of additional blur cues. Concussed participants showed reduced accommodative responses to a 0.1-Hz stimulus in monocular conditions, indicating mild accommodative deficits in the absence of disparity cues.

Each year, 50 million to 60 million people experience traumatic brain injury (TBI) globally, highlighting a significant public health concern.^1^ Mild TBI (mTBI), including concussions, represents over 90% of TBI cases and disproportionately affects children and adolescents.^1^ In the United States, 1 million to 2 million children and adolescents experience mTBI each year. Those with a history of concussion have a further fourfold risk of recurrent concussions^2^ which can result in increased symptomatology.^3^^,^^4^ Compared to adults, children and adolescents also tend to require longer recovery from concussions due to immaturity of the developing brain.^2^^,^^5^

Concussions are associated with wide-ranging and debilitating symptoms, which include headaches, nausea, fatigue, dizziness, vomiting, and cognitive deficits such as poor memory or attention.^6^^–^^9^ Oculomotor deficits are also prevalent following concussions, with patients often reporting blurred vision, eye fatigue, photophobia, diplopia, and difficulty with reading.^10^^–^^15^ In addition, many concussed individuals show difficulties in busy environments when switching focus from near to far targets or when tracking moving objects,^16^^,^^17^ pointing to deficits in dynamic visual processing. Associated oculomotor disorders are also frequently observed. In one study, 69% of adolescents 11 to 17 years of age had at least one vision disorder following concussion: 51% with accommodative dysfunction, 49% with convergence insufficiency, and 29% with saccadic dysfunction,^18^ reflecting rates that are significantly higher than those in the general population.^19^ Although most concussed patients recover within the first weeks after injury,^20^^,^^21^ an estimated 10% to 30% of adolescents experience post-concussion syndrome (PCS), whereby symptoms persist for longer than 4 weeks^21^^,^^22^ and may contribute to reduced quality of life.^23^^,^^24^ Oculomotor deficits represent a phenotypical subtype of PCS^10^ and have been reported in 88% of adolescents with PCS.^25^ However, current evidence is largely based on subjective patient reports and clinical measurements (e.g., push-up accommodative amplitude, near point of convergence),^11^^,^^12^^,^^14^^,^^18^^,^^22^ and the underlying mechanisms resulting in accommodative and vergence deficits remain unclear. Measurements that can capture both accommodative and vergence responses objectively and continuously at high sampling rates are needed to better quantify oculomotor responses. Such techniques are also critical to assessing the dynamic visual processing deficits that are often observed in concussions. Although several studies have demonstrated deficits in oculomotor dynamics in concussion using objective measurements,^26^^–^^30^ these studies have been restricted to adults^26^^,^^28^^,^^29^ or did not record accommodation and vergence in the same individual to inform about the interactive nature of these systems.^26^^–^^30^

The present study used eccentric photorefraction to objectively measure open- and closed-loop accommodative and vergence responses to a moving stimulus in a sample of symptomatic adolescents with a recent history of concussion (>4 weeks to <12 months) and a group of healthy controls. Participants viewed an age-appropriate movie binocularly (accommodation and vergence closed-loop) and monocularly (vergence open-loop, with disparity cues removed), as well as a Difference of Gaussians (DoG) target binocularly (accommodation open-loop, with blur cues removed as the DoG target contains only low spatial frequencies that are not cues to accommodation).^31^ Simultaneous measurements of accommodation and vergence responses were collected during stimulus presentations, and we hypothesized reduced response amplitudes in concussed compared to control participants.

## Methods

### Participants

Participants were recruited from patient and staff populations at Boston Children's Hospital Department of Ophthalmology, Stanford Children's Department of Ophthalmology, and Salus University's Pennsylvania of College of Optometry. Concussed individuals were recruited via referrals to vision providers, as well as from concussion clinics. Control participants included children of department staff, patients who came to eyecare clinics for routine eye exams, and individuals from the local community. Inclusion criteria were 8 to 18 years of age, habitual distance visual acuity of 20/25 or better Snellen equivalent in both eyes, and full ocular motility. Concussed participants were included if the concussion was diagnosed by a physician (in accordance with the Berlin Consensus Statement on Concussion in Sport)^32^ >4 weeks and >12 months from the time of the study visit. Concussed participants were included in the study if they were referred to an eye care provider for persistent concussion symptoms greater than 1 month, and reported symptoms on the Convergence Insufficiency Symptoms Survey on the day of the study visit.^33^ Exclusion criteria were history of amblyopia (≥2 lines interocular difference in best-corrected visual acuity at distance or near), strabismus, nystagmus, refractive surgery, in-office vision therapy, or ocular injury; constant or intermittent esotropia at distance or near; constant exotropia at distance or near; intermittent exotropia at distance; or vertical heterophoria ≥2 Δ at distance or near. Participants were also excluded if they were taking any medications or had any disease known to affect the oculomotor system or developmental delay that would interfere with testing. Cycloplegic refraction of participants without habitual correction was required to be <2.00 D hyperopia, <1.00 D myopia, <1.00 D anisometropia, and <1.25 D astigmatism. Participants with habitual correction were required to have hyperopic sphere power symmetrically reduced by no more than 1.50 D, spherical equivalent myopia and anisometropia within 1.00 D, astigmatism within 1.00 D, and axis within ±10° for cylinder power of ≥1.00 D. The study was conducted in accordance with the tenets of the Declaration of Helsinki and was approved by the institutional review boards of Boston Children's Hospital, Stanford University, and Salus University. Participants 18 years of age provided written informed consent prior to the study. Minors (<18 years) gave assent, and the accompanying parent or legal guardian provided written permission for the minor to take part in the study.

### Instrumentation

Measurements of accommodation and eye position were obtained using the PowerRef 3 (Plusoptix, Nuremberg, Germany) at a sampling rate of 50 hertz (Hz). The PowerRef 3 emits infrared (IR) light to create a luminance gradient profile across the pupil that indicates emmetropic, myopic, or hyperopic refractive estimates in the vertical meridian. The PowerRef 3 provides continuous monocular or binocular measurements of accommodative state in the vertical meridian (–7.00 to +5.00 D in 0.01-D steps), pupil size (3.0 to 8.0 mm in 0.1-mm steps), and eye position using Purkinje image eye-tracking (tracking range up to 11.52° × [pupil size] ± 2°).

### Experimental Set-Up

**Figure 1.**
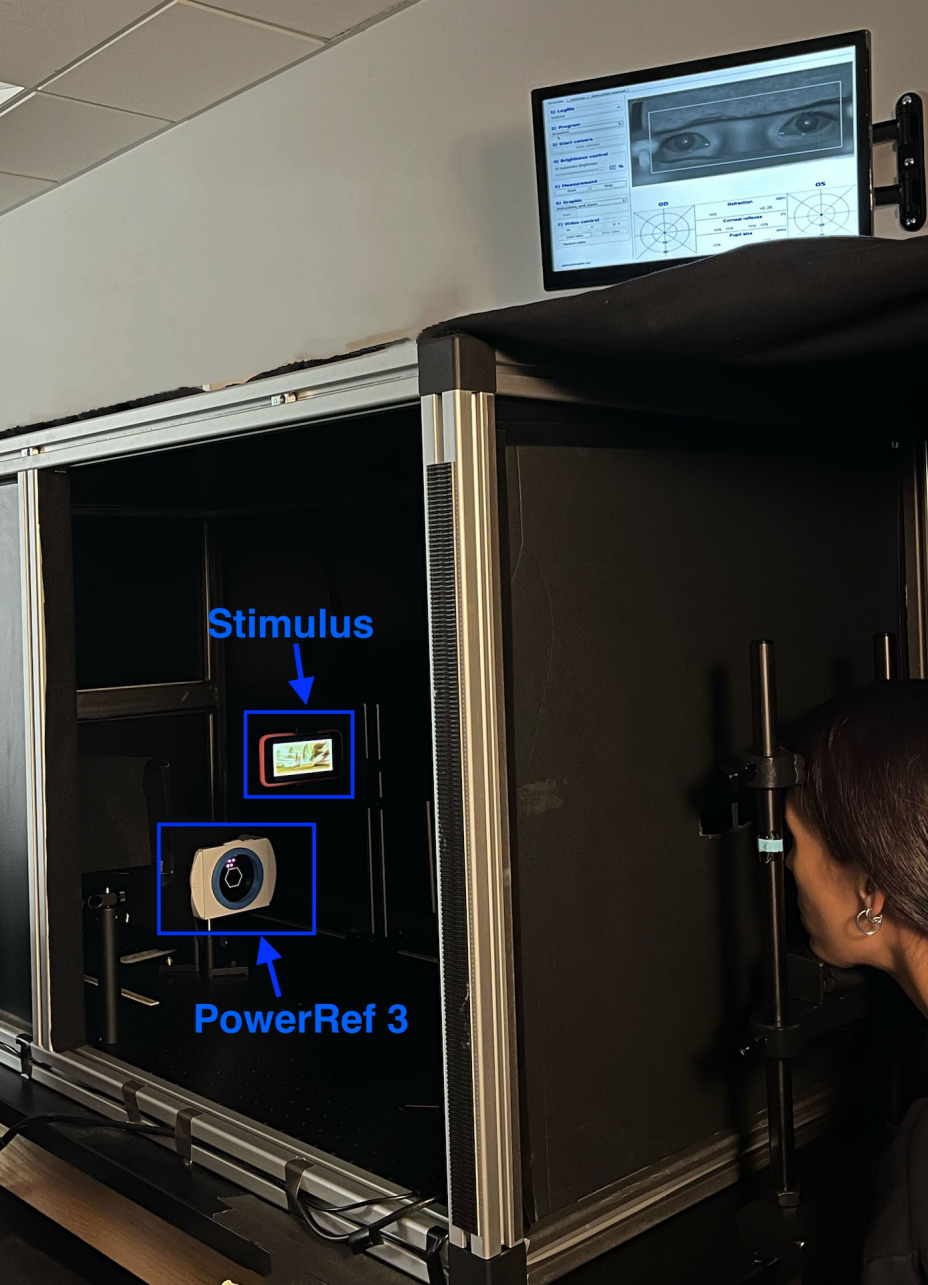
Visualization of experimental set-up. *Note:* Room lights were turned off and the light-shielding enclosure was fully covered during the study.

The experimental set-up is illustrated in Figure 1. Participants placed their head in a chin and forehead rest to minimize movements. Room lights were turned off to obtain optimal pupil readings. The study equipment was located inside an enclosure (see Fig. 1) to shield light and remove external visual distractors. Through a small aperture (100-mm width × 20-mm height), participants viewed an age-appropriate, high-contrast cartoon movie stimulus with a wide range of spatial frequencies or a DoG target on a 4-inch iPod Touch display (resolution, 1136 × 640 pixels; Apple Inc., Cupertino, CA, USA) in primary gaze. The DoG target was generated in MATLAB (MathWorks, Natick, MA, USA), and the resulting image was presented on the iPod Touch using Keynote presentation. To remove the appearance of edges of the iPod Touch display (i.e., blur cues), two layers of a gradient border target printed on transparency paper (i.e., two printed gradient borders) were placed over the display such that edges were smoothed out and not visible. The iPod Touch was mounted on a camera arm attached to a motorized track (Zaber Technologies, Vancouver, BC, Canada), which was controlled via an external Dell PC (Dell Technologies, Round Rock, TX, USA) running custom-written MATLAB scripts. Measurements of accommodation and eye position were continuously recorded using the PowerRef 3, which was positioned at 1-meter optical path length from the eyes. An IR-reflecting first surface mirror (102 mm × 127 mm) and a transparent hot mirror (45° angle of incidence, 101 mm × 127 mm) were used to reflect the IR light from the PowerRef 3 to the eyes. Additional lens calibrations were performed to obtain more accurate accommodative measurements (see Procedures for details).^34^^,^^35^ For such calibrations, the back panel of the enclosure was removed, and participants fixated a 20/150 single-surround letter displayed on an Apple iPad (resolution, 2048 × 1536 pixels) at a 3-meter viewing distance.

### Procedures

After providing written informed assent and consent, general eligibility criteria were confirmed, and a binocular vision examination was performed. This consisted of habitual monocular visual acuity at distance and near, stereopsis, pupillary examination, ocular alignment testing, near point of convergence, positive and negative fusional vergence ranges, vergence facility, accommodative amplitude, and monocular accommodative facility. Participants also underwent cycloplegic refraction (if not done by the clinician conducting the binocular vision exam within the last 6 months) at the end of the study visit to confirm eligibility.

### Objective Measurements of Accommodation and Vergence

Following the vision examination and a break of approximately 10 minutes to allow participant set-up for objective measurements, participants were informed that the movie stimulus or DoG target would move back and forth toward and away from them. In an effort to measure naturalistic vergence and accommodative responses, participants were simply instructed to look at the movie stimulus or DoG target. No further task instructions were given (i.e., participants were not asked to keep the stimulus clear or single). Participants viewed the movie stimulus binocularly (accommodation and vergence closed-loop) and monocularly (vergence open-loop), and the DoG target binocularly (accommodation open-loop). For monocular viewing, both the left and right eyes were tested, and the occluded eye was covered using an opaque IR-passing Kodak Wratten 89B filter (Kodak, Rochester, NY, USA). The order of viewing conditions (binocular-movie, monocular-movie, binocular-DoG) was counterbalanced across participants.

**Figure 2.**
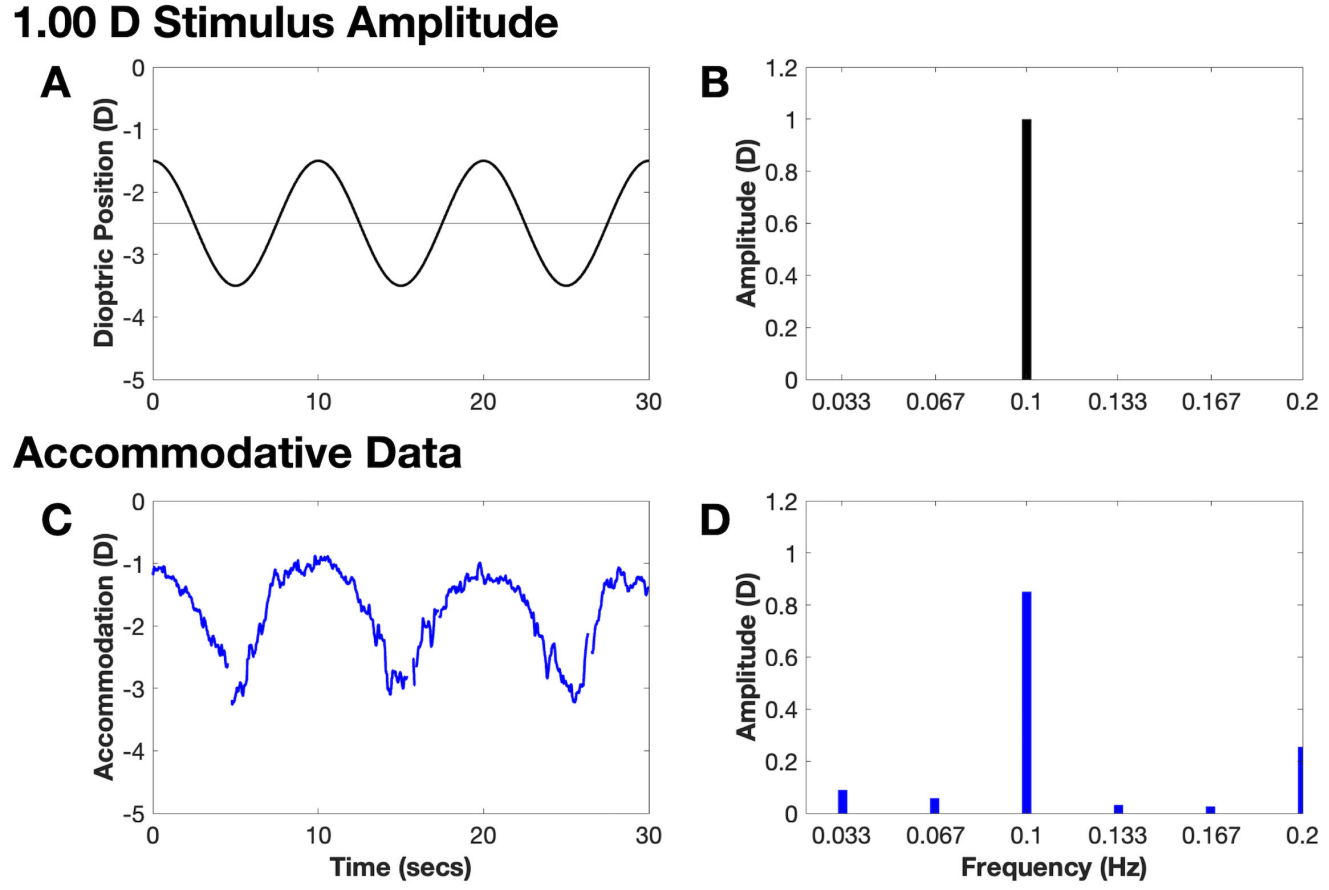
(**A**) Sinusoidal stimulus movement profile with 1.00-D amplitude and 2.50-D midpoint. (**B**) Corresponding amplitude spectra. (**C**) Accommodative response to the stimulus movement. (**D**) Response amplitude showing a peak at the 0.1-Hz stimulus frequency.

The movie stimulus and DoG target moved sinusoidally toward and away from the participant at four different dioptric stimulus amplitudes^36^ (1.50 D, 1.00 D, 0.50 D, and 0.25 D) with a midpoint of 2.50 D (Fig. 2). The stimulus amplitude refers to the distance between the midpoint and the nearest or farthest position (e.g., with a 1.50-D amplitude, the stimulus traveled a total of 3.00 D between the nearest and farthest position). Higher amplitudes, therefore, covered larger distances, resulting in the stimulus moving beyond the depth of field and creating retinal blur that stimulates an accommodative response.^37^ Conversely, lower amplitudes covered smaller distances, and the stimulus traveled within or only slightly beyond the depth of field, making it more difficult to detect movement. Each stimulus amplitude was presented once in each viewing condition. The stimulus was programmed to move for three cycles at a frequency of 0.1 Hz for a total trial duration of 30 seconds for each stimulus amplitude. Each cycle started at the far point location of the stimulus movement profile (see Fig. 2). The order of stimulus amplitudes was randomized across participants and remained identical across the different viewing/stimulus conditions for each participant. Each participant also underwent a lens calibration at the end of the study (plano to +4 D lenses in 1-D steps).^38^ Each lens was held in front of the calibrated eye for 5 seconds,^34^^,^^35^ and the opaque IR-passing Kodak Wratten 89B filter was additionally used to occlude the calibrated eye to avoid lens-induced accommodative responses. The non-calibrated eye fixated a 20/150 single-surround letter at a 3-meter viewing distance.

### Data Processing

#### Data Filtering

PowerRef 3 measurements of accommodation, pupil size, and eye position were extracted using MATLAB R2022a. Data were filtered offline to remove measurements outside the working range of the PowerRef 3. Measurements were removed if refractive values were <–6.00 D or >4.00 D,^39^ pupil size was <4 mm or >8 mm,^39^ and horizontal or vertical gaze position was ±10° of visual angle from the primary position.^40^ Samples were also removed if changes between two consecutive refractive measurements were >10 D/s to remove physiologically unlikely values.^41^ Three samples before and after a period of missing data (e.g., due to blinks) were additionally excluded to remove artifacts. The filtered, accommodative, and eye position measurements were then smoothed over a five-sample window using the moving average method. To obtain horizontal vergence measurements, the horizontal gaze positions (in degrees of visual angle) of the right eye (OD) were first subtracted from those of the left eye (OS). Using the interpupillary distance measurements provided by the PowerRef 3, the vergence data were then converted to meter angles (MA).

#### Data Quality Assessment

**Figure 3.**
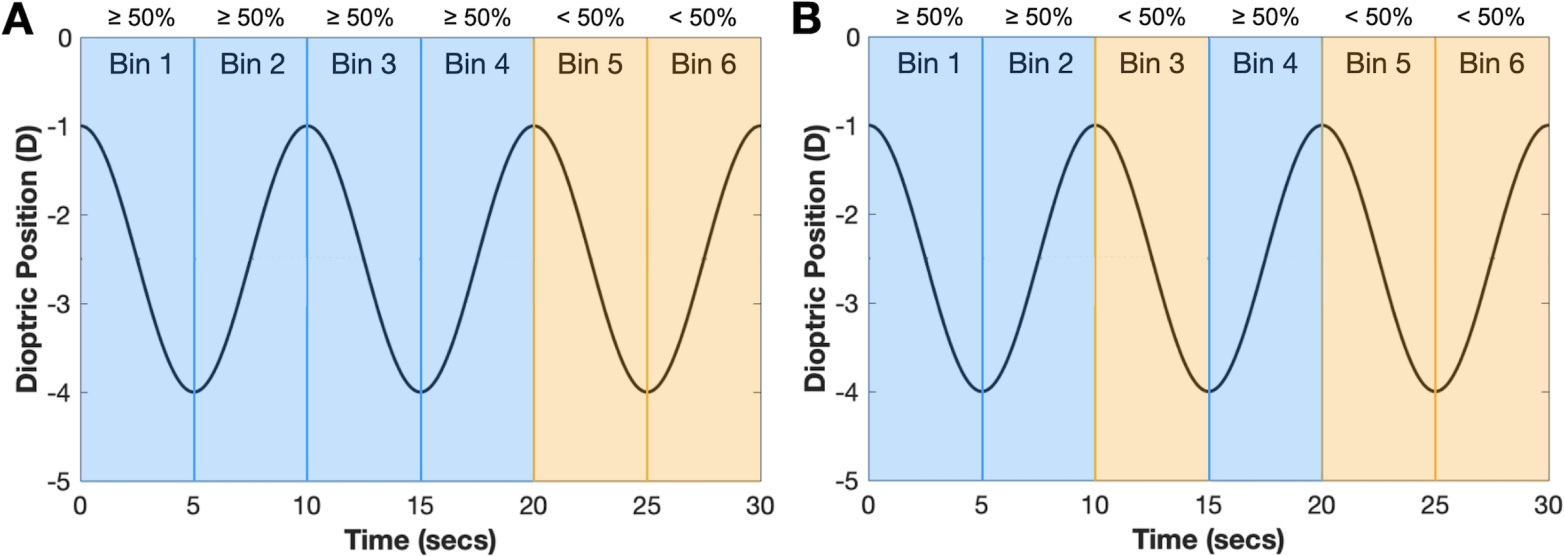
Simulated sinusoidal data (*black curve*) across a 30-second viewing period is divided into 5-second bins (bins 1 to 6), with each 10-second cycle consisting of two bins. The percentage of available data samples was calculated (with each bin consisting of a total of 250 possible data samples) and is indicated at the top of each bin (≥50% or <50 %). (**A**) The data quality criterion was met because two of three cycles had at least 50% data available (*blue*). (**B**) The data quality criterion failed because only one cycle has sufficient data (*blue*).

Experimental data were included in the statistical analysis if all quality criteria were met. Separately for each viewing condition (binocular-movie, monocular-movie, or binocular-DoG) and stimulus amplitude (1.50 D, 1.00 D, 0.50 D, or 0.25 D), accommodative and vergence measurements were first divided into 5-second bins (see Fig. 3). This resulted in a total of six bins (30-second stimulus duration/5-second bins), and each 10-second cycle consisted of two bins. The proportion of available data was then computed for each of the six bins, and those with ≥50% available data were flagged. The data quality criterion was met if at least two of three cycles contained only flagged bins (see Fig. 3). These data quality parameters were chosen to ensure that sufficient data were available across each cycle.

#### Lens Calibration

PowerRef 3 measurements for lens calibrations were filtered (see Data Filtering), and the mean refractive value for a given lens power was computed if ≥50% of the data (i.e., ≥2.5 seconds) was available for both eyes. Anisometropic differences were computed at each lens power, and a line function was fitted. Calibration functions were computed if usable data from at least four of the five powers were available, and slopes were obtained if the *R*^2^ value was ≥0.80. The experimental data were calibrated by applying individual slopes, or a group calibration factor (based on all participants with a valid calibration slope [*n* = 23], who were equally distributed across the concussed and control groups) was used if an individual slope was not available (OD_slope_ = 1.10, OS_slope_ = 1.04).

### Data Analysis

#### Group Differences in Dynamic Accommodation and Vergence

Fourier analysis was conducted to assess the accommodative and vergence response amplitudes at the 0.1-Hz stimulus frequency. Similar to the stimulus amplitude, the response amplitude refers to the magnitude between the dioptric midpoint and the nearest or farthest position of the accommodative/vergence response (e.g., a 0.80-D accommodative response amplitude suggests that the participant had a total change in accommodation of 1.60 D from the nearest to the farthest position). Missing accommodative or vergence data points were linearly interpolated, and Fourier analysis was performed using the MATLAB fft function (i.e., fast Fourier transform). The magnitude of the response amplitude at the signal frequency (0.1 Hz) was obtained (see Fig. 2).

For statistical analysis of accommodation, data from the eye with the better monocular accommodative facility (MAF) was used. If MAF was equal for both eyes, data from the right eye were used. A separate two-way repeated-measures analysis of variance (ANOVA) was conducted for each stimulus amplitude (1.50 D, 1.00 D, 0.50 D, and 0.25 D) to assess the effects of group (control, concussed) and viewing condition (binocular, monocular, DoG) on accommodative and vergence response amplitudes at the 0.1-Hz signal frequency. All data analysis was performed using R 4.1.2 (R Foundation for Statistical Computing, Vienna, Austria).

## Results

### Participants

Thirty-seven concussed participants and 34 healthy controls took part in the study. Five of the concussed participants and two of the controls were enrolled in the study but excluded from analysis due to equipment failure resulting in data loss (*n* = 1), inability to fixate stimuli due to extreme fatigue (*n* = 1), withdrawal from study after the eye exam (*n* = 1), not meeting eligibility criteria based on distance visual acuity (*n* = 3), or the presence of esotropia (*n* = 1). This resulted in a final sample size of 32 concussed (23 females, nine males; mean age, 14.4 ± 2.6 years; mean number of days since concussion, 107 ± 48 days; range, 36–273 days) and 32 healthy controls (18 females, 14 males; mean age, 12.7 ± 2.1 years). The causes of concussion included sports-related activities (22 of 32, 69%), motor vehicle accidents (1 of 32, 3%), and other causes such as falls (9 of 32, 28%). Participants were Asian (concussed: *n* = 3, 9.4%; controls: *n* = 24, 75.0%) or White (concussed: *n* = 26, 81.3%; controls: *n* = 6, 18.8%), or they indicated more than one race (concussed: *n* = 1, 3.1%; controls: *n* = 2, 6.3%; race unknown: *n* = 2). Most participants were non-Hispanic/Latino (concussed: *n* = 28, 87.5%; controls: *n* = 31, 96.9%), and few participants were Hispanic/Latino (concussed: *n* = 3, 9.4%; controls: *n* = 1, 3.1%; ethnicity unknown: *n* = 1). Race and ethnicity were self-reported.

### Data Availability

For accommodation, complete datasets, which included data in the binocular, monocular, and DoG viewing conditions and for all stimulus amplitudes (1.50 D, 1.00 D, 0.50 D, and 0.25 D), were obtained in 24 of 32 concussed (75.0%) and 17 of 32 control participants (53.1%). In addition, three concussed (9.4%) and 10 control participants (31.3%) had accommodative data for all stimulus amplitudes in two of the three viewing conditions (and in most cases also usable data for the third viewing condition).

For vergence, complete datasets were available for 17 of 32 concussed participants (53.1%) and 14 of 32 control participants (43.8%). Eight concussed participants (25.0%) and seven control participants (21.9%) had vergence data for all stimulus amplitudes in two of the three viewing conditions. The remaining participants (included for analysis) provided accommodative or vergence data in stimulus amplitudes across viewing conditions. Missing data were primarily due to short periods of data loss during recordings or after filtering such that the number of data samples was insufficient for analysis (see Data Processing).

### Accommodation: Response Amplitudes

**Figure 4.**
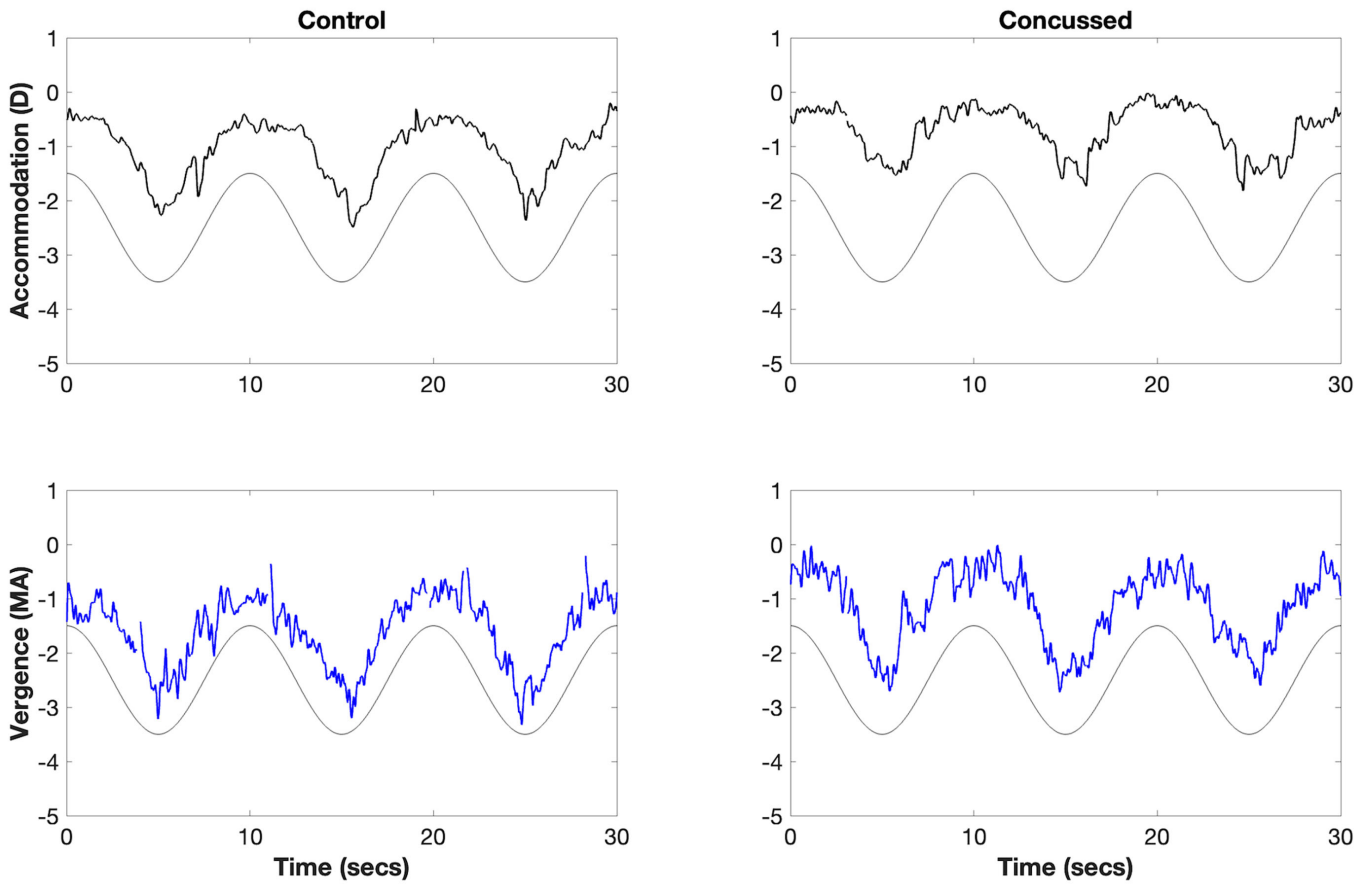
Smoothed (over five data samples) accommodative (*top*) and vergence (*bottom*) responses from a control participant (*left*) and a concussed participant (*right*) for a stimulus movement profile with 1.00-D amplitude in the binocular condition. Accommodative lags and associated vergence disparity were observed.

**Figure 5.**
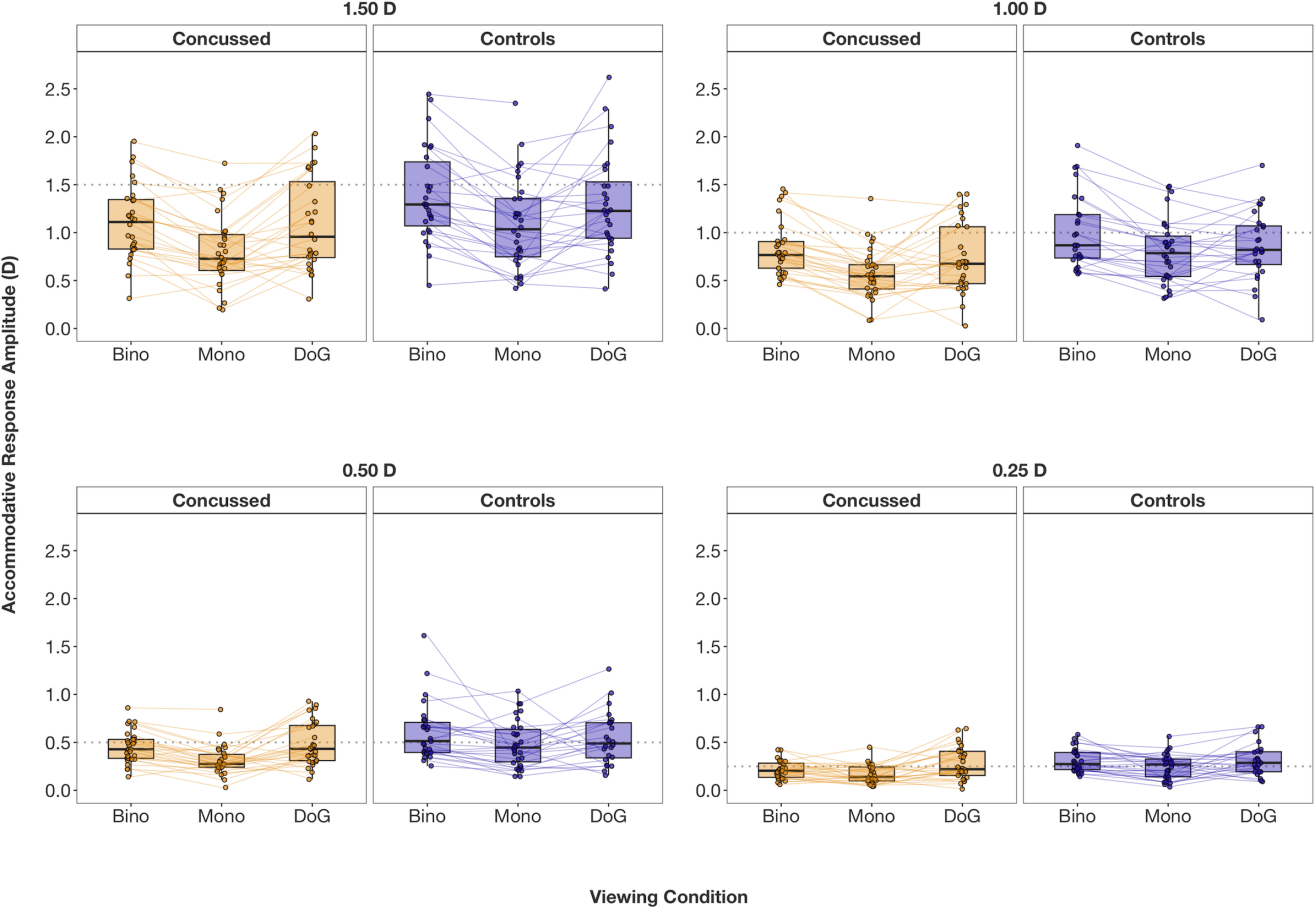
Distribution of accommodative response amplitudes (in diopters) at each stimulus amplitude (*dotted lines*; 1.50 D, 1.00 D, 0.50 D, 0.25 D), separately for concussed and control participants as well as viewing condition. Bino, binocular; Mono, monocular. Connecting lines indicate related data points from the same participant.

Accommodative response amplitudes are visualized in Figures 4 and 5, and descriptive and inferential statistics are summarized in Tables 1 and 2. The 2 (group: control, concussed) × 3 (viewing condition: binocular, monocular, DoG) ANOVA showed no significant interactions at any stimulus amplitude: at 1.50 D, *F*(1.64, 78.74) = 0.26, *P* = 0.727; at 1.00 D, *F*(1.51, 72.52) = 1.23, *P* = 0.289; at 0.50 D, *F*(2, 86) = 1.74, *P* = 0.182; at 0.25 D, *F*(1.67, 80.05) = 1.24, *P* = 0.290. A significant main effect of group was revealed for the smaller stimulus amplitudes—at 0.50 D, *F*(1, 43) = 2.62, *P* = 0.055; at 0.25 D, *F*(1, 48) = 4.53, *P* = 0.039—indicating slightly reduced accommodative responses in concussed participants overall. A similar, albeit not significant, trend was observed for the larger stimulus amplitudes: at 1.50 D, *F*(1, 48) = 2.59, *P* = 0.114; at 1.00 D, *F*(1, 48) = 2.62, *P* = 0.112. Post hoc *t*-tests with a Bonferroni-adjusted alpha level of 0.017 showed significantly reduced accommodative responses in concussed participants compared to controls during monocular viewing for all stimulus amplitudes (all *P* < 0.012) (see Table 1 for statistical values). Accommodative responses were also reduced in concussed participants during binocular viewing, although only significantly reduced at the smallest 0.25-D stimulus amplitude (*P* = 0.001). No significant group differences in accommodative responses were found for the DoG target (all *P* *>* 0.05) (see Table 1).

**Table 1.** Statistical Values for Response Amplitudes of Accommodation and Vergence for Each Viewing Condition

		Accommodation (D)					Vergence (MA), Mean ± SD^*^	
Stimulus Amplitude	Viewing Condition	Control, Mean ± SD	Concussed, Mean ± SD	*t*	*df*	*P*	Control	Concussed
1.50 D	Bino	1.40 ± 0.49	1.11 ± 0.39	–2.40	49.42	0.020	1.26 ± 0.19	1.18 ± 0.21
	Mono	1.09 ± 0.47	0.80 ± 0.36	–2.63	54.49	0.011	0.52 ± 0.17	0.44 ± 0.21
	DoG	1.29 ± 0.52	1.09 ± 0.48	–1.50	53.61	0.139	1.15 ± 0.21	1.16 ± 0.21
1.00 D	Bino	1.01 ± 0.39	0.83 ± 0.28	–1.95	44.98	0.058	0.91 ± 0.16	0.88 ± 0.13
	Mono	0.80 ± 0.34	0.57 ± 0.26	–2.96	54.72	0.005	0.37 ± 0.16	0.29 ± 0.12
	DoG	0.86 ± 0.35	0.73 ± 0.36	–1.33	52.57	0.189	0.82 ± 0.16	0.79 ± 0.23
0.50 D	Bino	0.61 ± 0.30	0.45 ± 0.17	–2.35	39.37	0.024	0.51 ± 0.06	0.48 ± 0.08
	Mono	0.49 ± 0.24	0.32 ± 0.16	–3.23	49.74	0.002	0.20 ± 0.10	0.16 ± 0.06
	DoG	0.54 ± 0.28	0.49 ± 0.23	–0.77	44.83	0.443	0.44 ± 0.12	0.49 ± 0.12
0.25 D	Bino	0.31 ± 0.12	0.21 ± 0.09	–3.38	48.69	0.001	0.25 ± 0.06	0.25 ± 0.05
	Mono	0.25 ± 0.13	0.17 ± 0.10	–2.67	55.54	0.010	0.14 ± 0.08	0.13 ± 0.07
	DoG	0.32 ± 0.16	0.28 ± 0.17	–0.92	51.39	0.364	0.23 ± 0.08	0.26 ± 0.09

*df*, degrees of freedom, Bino, binocular; Mono, monocular.

* Only descriptive statistics are reported for vergence because no significant differences were found between the concussed and the control groups.

**Table 2.** Statistical Values for Response Amplitudes of Accommodation and Vergence for Contrasting Viewing Conditions

		Accommodation (D)								
		Control			Concussed			Vergence (MA)^*^		
Stimulus Amplitude	Viewing Condition	*t*	*df*	*P*	*t*	*df*	*P*	*t*	*df*	*P*
1.50 D	Bino vs. mono	6.26	25	<0.001	6.70	26	<0.001	20.24	48	<0.001
	Bino vs. DoG	0.97	23	0.342	0.42	25	0.675	2.03	46	0.048
	Mono vs. DoG	2.94	26	0.007	4.41	27	0.007	20.88	47	<0.001
1.00 D	Bino vs. mono	4.94	24	<0.001	7.74	28	<0.001	16.94	45	<0.001
	Bino vs. DoG	2.01	21	0.058	1.16	27	0.257	2.11	44	0.040
	Mono vs. DoG	1.25	25	0.222	2.61	28	0.014	14.11	44	<0.001
0.50 D	Bino vs. mono	2.75	25	0.011	6.09	26	<0.001	20.11	43	<0.001
	Bino vs. DoG	1.43	20	0.170	–1.68	26	0.106	1.80	45	0.079
	Mono vs. DoG	1.67	22	0.110	4.48	25	<0.001	13.75	39	<0.001
0.25 D	Bino vs. mono	3.52	26	0.002	1.93	27	0.064	10.08	49	<0.001
	Bino vs. DoG	–1.11	22	0.912	–2.65	27	0.013	0.49	47	0.628
	Mono vs. DoG	2.59	24	0.016	3.43	26	0.002	7.12	44	<0.001

* For vergence, *t*-test results are reported collapsed across control and concussed participants because no significant differences were found between the control and concussed groups.

A significant main effect of viewing condition was additionally found across all stimulus amplitudes: at 1.50 D, *F*(1.64, 78.74) = 24.96, *P* < 0.001; at 1.00 D, *F*(1.51, 72.52) = 19.10, *P* < 0.001; at 0.50 D, *F*(2, 86) = 14.89, *P* < 0.001; at 0.25 D, *F*(1.67, 80.05) = 10.94, *P* < 0.001. Post hoc paired *t*-tests were conducted separately in controls and concussed participants, given the group effect at smaller stimulus amplitudes (and the trend in the same direction at larger stimulus amplitudes; Bonferroni-adjusted alpha level of 0.008). For both groups, accommodative responses were significantly greater under binocular than monocular viewing conditions across all stimulus amplitudes (*P* *<* 0.003) (see Table 2 for statistical values), except at the 0.50-D and 0.25-D amplitudes in controls and concussed participants, respectively (*P* *>* 0.05) (see Table 2). In concussed participants, accommodative responses were also significantly greater when viewing the DoG target compared to the monocular condition (*P* < 0.008) (Table 2), except at the 1.00-D stimulus amplitude (*P* = 0.014). In control participants, only the 1.50-D stimulus amplitude showed significantly higher accommodative responses for the DoG target compared to the monocular condition (*P* = 0.007) (Table 2). No significant differences in accommodative responses were found for either group when comparing the DoG and binocular conditions (all *P* *>* 0.008) (Table 2).

In summary, accommodative responses were greater under binocular conditions (accommodation and vergence closed-loop) than monocular conditions (vergence open-loop) in both groups. No significant differences were found between binocular (accommodation and vergence closed-loop) and DoG conditions (accommodation open-loop). In concussed participants, accommodative responses were overall higher for DoG than monocular conditions. In controls, only the largest 1.50-D stimulus amplitude showed this pattern, but no differences in accommodative responses were found at other stimulus amplitudes.

### Vergence: Response Amplitudes

**Figure 6.**
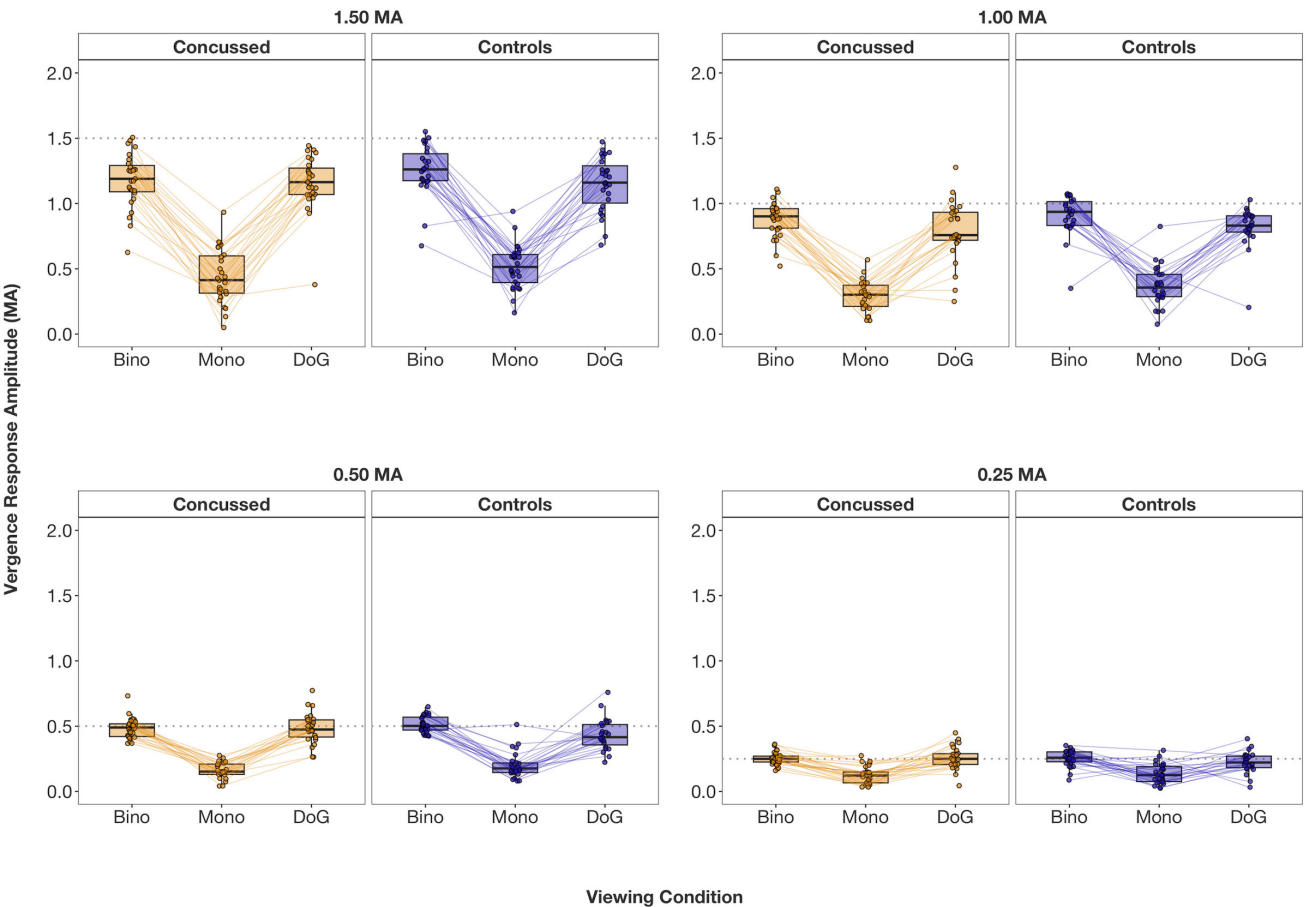
Distribution of vergence response amplitudes (in meter angles) at each stimulus amplitude (*dotted lines*; 1.50 MA, 1.00 MA, 0.50 MA, 0.25 MA), separately for concussed and control participants as well as viewing condition. Connecting lines indicate related data points from the same participant.

Vergence response amplitudes are visualized in Figures 4 and 6, and descriptive and inferential statistics are summarized in Tables 1 and 2. The 2 (group: control, concussed) × 3 (viewing condition: binocular, monocular, DoG) ANOVA on response amplitudes for vergence showed no significant interactions for any stimulus amplitude: at 1.50 MA, *F*(2, 84) = 0.99, *P* = 0.377; at 1.00 MA, *F*(2, 78) = 0.25, *P* = 0.783; at 0.50 MA, *F*(2, 72) = 0.81, *P* = 0.447; at 0.25 MA, *F*(2, 84) = 1.79, *P* = 0.174. Unlike response amplitudes for accommodation, no significant main effects of group were revealed for vergence: at 1.50 MA, *F*(1, 42) = 1.76, *P* = 0.192; at 1.00 MA, *F*(1, 39) = 3.03, *P* = 0.090; at 0.50 MA, *F*(1, 36) = 0.53, *P* = 0.473; at 0.25 MA, *F*(1, 42) = 0.41, *P* = 0.528. A significant main effect of viewing condition was found across all stimulus amplitudes: at 1.50 MA, *F*(2, 84) = 259.48, *P* < 0.001; at 1.00 MA, *F*(2, 78) = 123.33, *P* < 0.001; at 0.50 MA, *F*(2, 72) = 171.27, *P* < 0.001; at 0.25 MA, *F*(2, 84) = 41.16, *P* < 0.001. Post hoc paired *t*-tests were conducted using a Bonferroni-adjusted alpha level of 0.008. Vergence responses were significantly higher under binocular than monocular viewing conditions across all stimulus amplitudes (all *P* *<* 0.001) (see Table 2). There was no significant difference between the binocular and DoG conditions (all *P* > 0.040) (Table 2). However, vergence responses were significantly higher for DoG compared to monocular conditions (all *P* < 0.001). In summary, vergence responses were higher under binocular (accommodation and vergence closed-loop) and DoG conditions (accommodation open-loop) than monocular conditions (vergence open-loop).

### Control Experiment: Monocular DoG

An additional six control participants without a history of concussion (four females, two males; mean age, 12.5 ± 2.7 years; age range, 9–15 years) were tested under monocular DoG and binocular DoG viewing conditions to confirm that the DoG stimulus appropriately induced open-loop accommodation. Reduced accommodative response amplitudes in the monocular compared to binocular DoG condition would also point to a significant role of disparity cues in driving accommodation in the current paradigm. The same procedures were applied as in the main experiment (see Data Processing and Data Analysis), except that the participant's right eye was selected for statistical analysis. Paired *t-*tests were used to compare response amplitudes in the binocular and monocular DoG conditions.

In the control experiment, accommodative response amplitudes were significantly reduced in the monocular DoG compared to the binocular DoG condition at the 1.50-D and 1.00-D stimulus amplitudes: at 1.50 D, monocular = 0.29 ± 0.21 D versus binocular = 0.90 ± 0.37 D, *t*(5) = 4.07, *P* = 0.010; at 1.00 D, monocular = 0.22 ± 0.10 D versus binocular = 0.68 ± 0.27 D, *t*(5) = 4.72, *P* = 0.005. Accommodative response amplitudes were also reduced in the monocular DoG conditions at the smaller 0.50-D and 0.25-D stimulus amplitudes, but these differences were not significant: at 0.50 D, monocular = 0.30 ± 0.17 D versus binocular = 0.39 ± 0.17 D, *t*(5) = 1.22, *P* = 0.277; at 0.25 D, monocular = 0.17 ± 0.12 D versus binocular = 0.20 ± 0.06 D, *t*(5) = 0.99, *P* = 0.367.

Vergence response amplitudes were also significantly lower in the monocular DoG compared to the binocular DoG condition, except at the lowest 0.25-MA stimulus amplitude: at 1.50 MA, monocular = 0.29 ± 0.19 MA versus binocular = 1.24 ± 0.07 MA, *t*(4) = 8.45, *P* = 0.001; at 1.00 MA, monocular = 0.21 ± 0.13 MA versus binocular = 0.76 ± 0.19 MA, *t*(5) = 8.73, *P* < 0.001; at 0.50 MA, monocular = 0.14 ± 0.10 MA versus binocular = 0.43 ± 0.12 MA, *t*(5) = 4.33, *P* = 0.008; at 0.25 MA, monocular = 0.12 ± 0.13 MA versus binocular = 0.20 ± 0.09 MA, *t*(5) = 1.23, *P* = 0.273.

## Discussion

The current study examined closed- and open-loop accommodative and vergence responses to a moving stimulus in symptomatic concussed adolescents and similarly aged controls. Our findings demonstrated differential response amplitudes depending on viewing condition and revealed mildly reduced accommodative response amplitudes in concussed participants.

In both groups, accommodative and vergence response amplitudes were greater and corresponded more closely to the stimulus demands under binocular conditions when both accommodation and vergence were closed-loop compared to monocular conditions when vergence was open-loop. As expected, the presence of disparity cues in the binocular condition therefore improved both accommodative and vergence responses to the moving stimulus.^42^ Although accommodative responses at the smaller 0.50 D and 0.25 D stimulus amplitudes did not significantly differ between binocular and monocular conditions, this was most likely due to the stimulus moving within or only slightly beyond the depth of field. Accommodative and vergence response amplitudes also tended to be greater in binocular conditions (accommodation and vergence closed-loop) when all visual cues were available, compared to the DoG conditions (accommodation open-loop) when accommodative cues were removed. However, no significant differences were found between the binocular and DoG conditions. This suggests that, although the additional blur cues in the binocular condition slightly improved accommodative response amplitudes, disparity cues primarily drove both accommodative and vergence responses to the moving target. This was further confirmed in the control experiment, with accommodative response amplitudes significantly reduced when disparity cues were removed under monocular DoG viewing conditions. Given that the stimulus was moving toward and away from participants, other cues such as proximal accommodation may have additionally impacted visual responses, but such cues would be consistent across viewing conditions. As expected, vergence response amplitudes were greater in the disparity-only DoG condition compared to the blur-only monocular condition. However, accommodative response amplitudes were also higher in the DoG than in the monocular condition at all stimulus amplitudes for concussed participants, and at the largest 1.50 D stimulus amplitude for controls. This may suggest a role of convergence accommodation, whereby disparity cues in the DoG condition facilitated accommodative responses in the current paradigm. Our findings therefore indicate that viewing conditions with vergence closed-loop and accommodation open-loop still result in appropriate accommodative responses; however, when vergence is open-loop and accommodation is closed-loop, vergence responses were significantly reduced. This suggests that both accommodation and vergence were highly driven by disparity cues and not blur cues when a stimulus is moving. Our findings are consistent with other studies demonstrating that disparity cues largely drive accommodative responses to moving targets.^43^^,^^44^ We further hypothesize that blur cues may be more critical when viewing static targets,^42^ and future work will be required to investigate the role of disparity and blur cues in accommodative responses to moving versus static targets.

Furthermore, no significant group differences were found for vergence or accommodative response amplitudes in the binocular and DoG conditions, although a trend for reduced accommodative response amplitudes was observed, and the 0.25-D stimulus amplitude resulted in a significant group difference for binocular conditions. This was likely a result of reduced variability in the responses such that smaller group differences can be detected. However, concussed participants had reduced accommodative response amplitudes in the monocular condition, pointing to a small accommodative deficit in the absence of disparity cues. These mild deficits in accommodation, but not vergence, are consistent with patients reporting blurred vision more frequently than diplopia following concussion^22^^,^^45^ (60% and 28%, respectively, after 1 month following concussion) and reflect the high prevalence of accommodative disorders observed clinically post-concussion.^11^^,^^26^^,^^46^^,^^47^ Our findings further showed no group differences in vergence response amplitudes, in contrast with clinical measurements whereby 60% of concussed adolescents^46^ present with receded near point of convergence (NPC).^27^^,^^48^^,^^49^ At the time of the study visit, 66% of concussed participants (21 of 32) in the current study presented with a receded NPC and 63% (20 of 32) had abnormal positive fusional vergence, reflecting rates similar to those in previous clinical reports.^27^^,^^48^^,^^49^ This indicates that the absence of any group differences in vergence response amplitudes could not be explained by concussed participants having normal vergence based on clinical tests. The discrepancy between the observed vergence response amplitudes and our clinical data may be explained by the higher viewing demand and therefore increased vergence effort during NPC measurements (normal cut-off point at 6 cm) compared to the current study (highest demand at 25 cm). Future work could explore associations between stimulus demand and vergence deficits in concussion by objectively measuring responses at closer viewing distances. Future studies could also investigate differences in accommodative responses when the stimulus is moving toward versus away from the participant, corresponding to plus and minus lenses in clinical accommodative facility testing, given that concussed patients have been shown to have greater difficulty clearing plus lenses than minus lenses.^46^

Several studies using objective measurements with comparable stimulus demands have previously been conducted to characterize accommodative or vergence responses in concussion.^26^^–^^30^ Participants were asked to repeatedly switch monocular focus (to assess accommodation) or binocular focus (to assess vergence) between two target positions (e.g., at 25 cm and 50 cm).^26^^,^^28^ Across all studies, concussed participants showed delayed and slowed accommodative or vergence responses.^26^^–^^30^ However, findings on response amplitudes were more variable: Consistent with our findings, Szymanowicz and colleagues^29^ found no differences between concussed and controls in vergence response amplitudes, whereas other studies have found group differences in accommodative response amplitudes^26^^,^^28^ or reduced vergence response amplitudes.^27^^,^^30^ This inconsistency between past and present findings is likely due to various methodological differences, including participant age (pediatric or adult) and time since concussion (weeks or years). The use of step changes in stimulus demand in previous studies also differs from the current paradigm, which used a sinusoidal movement profile that required participants to continuously track the target. It is possible that the continuous movement profiles in the current study facilitated vergence responses by keeping the target within Panum's fusional area.^50^ In contrast, step changes abruptly move the target outside Panum's fusional area and may require target prediction. Because target prediction has been shown to be affected in concussed participants,^51^ this could explain the reduced vergence responses observed in previous studies. Future studies could examine possible differences in smooth versus step vergence performance in concussion.

The current study has several limitations. Concussed individuals were recruited from vision examinations after being referred due to persistent symptoms, resulting in a sample of visually symptomatic participants who may not be representative of all patients with PCS. Our concussed participants may have also been frequently questioned about their vision symptoms, giving rise to potential voluntary or practice factors that could have affected accommodative responses. However, our objective measurements would minimize this possibility, and the inclusion of a control group would minimize the impact of such factors. Given our aim to better understand post-concussion oculomotor deficits, it is critical to examine accommodative and vergence responses specifically in patients reporting visual symptoms. Future studies should nevertheless also investigate accommodative and vergence responses in asymptomatic concussed individuals and explore the associations between visual symptoms and oculomotor performance. For example, it could be investigated whether concussed patients reporting higher levels of visual symptoms may be overexerting their vergence system to overcome accommodative deficits (i.e., convergence accommodation) or their accommodative system to overcome vergence deficits (i.e., accommodative convergence). A further limitation relates to the 0.1-Hz stimulus frequency used in the current study, which was kept constant across the different stimulus amplitudes. Although this allowed for comparability in our analysis, future work should disentangle stimulus speed and size of displacement to clarify whether the observed oculomotor responses at higher stimulus amplitudes are due to increased speed or larger stimulus displacements.

In conclusion, the present study objectively measured both dynamic accommodative and vergence responses in symptomatic concussed adolescents and found mild accommodative but not vergence deficits in response to a moving stimulus between 25 cm and 1 meter. Future studies will be necessary to identify and quantify the various methodological and contextual factors—such as stimulus movement profiles, participant age, or fatigue effects—to better understand the factors contributing to the observed oculomotor deficits following concussion.
